# Altered microstructure rather than morphology in the corpus callosum after lower limb amputation

**DOI:** 10.1038/srep44780

**Published:** 2017-03-17

**Authors:** Zhichao Li, Chuanming Li, Lingzhong Fan, Guangyao Jiang, Jixiang Wu, Tianzi Jiang, Xuntao Yin, Jian Wang

**Affiliations:** 1 Department of Radiology, Southwest Hospital, Third Military Medical University, Chongqing 400038, China; 2 National Laboratory of Pattern Recognition, Institute of Automation, Chinese Academy of Sciences, Beijing 100190, China; 3 Department of Rehabilitation, Southwest Hospital, Third Military Medical University, Chongqing 400038, China

## Abstract

The corpus callosum (CC) has been implicated in the reorganization of the brain following amputation. However, it is unclear which regions of the CC are involved in this process. In this study, we explored the morphometric and microstructural changes in CC subregions in patients with unilateral lower limb amputation. Thirty-eight patients and 38 age- and gender-matched normal controls were included. The CC was divided into five regions, and the area, thickness and diffusion parameters of each region were investigated. While morphometric analysis showed no significant differences between the two groups, amputees showed significant higher values in axial diffusivity, radial diffusivity and mean diffusivity in region II of the CC, which connects the bilateral premotor and supplementary motor areas. In contrast, the mean fractional anisotropy value of the fibers generated by these cortical areas, as measured by tractography, was significantly smaller in amputees. These results demonstrate that the interhemispheric pathways contributing to motor coordination and imagery are reorganized in lower limb amputees.

## Introduction

Amputation in humans has been reported to lead to extensive brain reorganization in the motor and somatosensory areas^1^,^2^,^3^. Expanded activation^4^,^5^,^6^ but reduced interhemispheric functional connectivity^7^ during sensory stimulation or movement was detected in these cortices. This has been interpreted as maladaptive plasticity in the representation of the former limb, triggered by loss of sensory inputs^4^ or voluntary control^6^. However, most of these studies included only patients with upper limb amputation, and the patterns of brain reorganization might vary significantly in lower limb amputees due to differentiated functions and representations between the upper and lower limbs. In addition, considering that the strength of interhemispheric functional connectivity between the homotopic regions is mediated by their structural connectivity^8^, it would be natural to postulate an association between the cortical regions with aberrant interhemispheric functional connectivity and the relevant commissural fibers in amputees, which could provide new insights into novel mechanisms of brain reorganization following amputation.

The corpus callosum (CC) is the largest white matter (WM) structure in the brain. It is known to harmonize interhemispheric function and is essential for the integration of perceptual, motor and other volitional processes^9^. Previous studies have established a topographical distribution of fiber connections to the different functional regions in midsagittal section of the CC^10^,^11^, and alterations in mid-sagittal CC area and thickness are the easiest to detect in terms of morphological changes in the CC. Neuroimaging evidence has revealed morphological abnormalities of the CC in patients with movement disorders, such as Huntington’s disease^12^ and motor coordination disorder^13^. In addition, the CC also demonstrates substantial inter-individual variability in local morphology among healthy individuals^14^, and can reorganize during perceptual deprivation^15^ or kinematic training^16^,^17^. However, it remains unknown whether morphological differences in the CC are evident after lower limb amputation. Because regions of the CC could reflect homotopic connections between the two hemispheres, changes to specific subregions might explain specific functional deficits in amputees, such as degradation of motor coordination. Accordingly, the CC can serve as a target for potential treatment^18^ and evaluation of functional rehabilitation.

Diffusion tensor imaging (DTI) has become an unrivalled technique for the study of structural connectivity and has been successfully utilized to identify WM plasticity in the adult brain^19^,^20^. Fractional anisotropy (FA) and mean diffusivity (MD) are the two most commonly used indices. FA is defined as the coefficient of variation of the eigenvalues, and its reduction implies decreased WM tract integrity, while MD is the average of the three eigenvalues of the diffusion tensor and measures the magnitude of water molecule diffusion. In addition, diffusion tractography techniques enable the examination of specific fiber connections. DTI studies have reported FA reductions in several areas after the amputation of a lower limb, including the prefrontal WM, which connects the bilateral premotor cortices^21^, or the callosal fibers projecting to the primary sensory cortex^4^. Discrepancies in the literature might be attributed to sample size or clinical heterogeneity, such as varying degrees of prosthesis use and time spans since amputation, which raise a fundamental issue that requires further investigation.

We hypothesized that: (1) specific CC subregions are involved in brain reorganization following lower limb amputation, and (2) WM fiber tracts passing through these specific subregions would also be disturbed. In the present study, the CC was segmented based on canonical methods and morphological measurements (callosal area and thickness) were performed. In addition, the FA value was calculated for each subregion using DTI data. To pinpoint the nature of diffusion changes, complementary DTI parameters such as axial diffusivity (AD, parallel diffusivity along the fiber bundles) and radial diffusivity (RD, average diffusivity in the plane perpendicular to the fiber bundles) were also investigated. Furthermore, fiber tracts passing through the affected subregions of the CC were visualized using diffusion tractography. Finally, the relationship between the imaging measures and clinical variables was investigated.

## Methods

### Subjects

Thirty-eight adult patients (28 male and 10 female) with unilateral lower limb amputation (16 patients on the left side and 22 on the right side) were recruited consecutively and prospectively from the Prosthetic and Orthotic Clinics at the Department of Rehabilitation, Southwest Hospital in Chongqing between December 2012 and December 2015. Sixteen amputations occurred at the transfemoral and 22 at the transtibial levels. All patients had been fitted with prostheses. Twenty-four patients underwent amputation following a traumatic injury, and the other amputations were due to tumors (two melanoma, five osteosarcoma) or osteomyelitis seven). Phantom limb pain (PLP) and stump pain were assessed by the five-category verbal rating scale^22^. Exclusion criteria were the following: (1) age at amputation or magnetic resonance imaging (MRI) scanning of less than 18 years or more than 60 years old, (2) amputation at another part of the body, (3) history of brain injury due to trauma, (4) presence of psychiatric or neurological illnesses and (5) duration between amputation and MRI scanning of less than one month.

Thirty-eight age- and sex-matched healthy controls without neurological or psychiatric diseases and with normal brain MRI (no brain atrophy, tumor, ischemia, hemorrhage or congenital abnormalities) were recruited from the local community. All participants were dominantly right-handed as determined by the Edinburgh Handness Inventory^23^ and had a score of 27 or higher on the Chinese version of the Mini-Mental Status Examination (MMSE)^24^. The study was conducted according to the Declaration of Helsinki and was approved by the Medical Research Ethics Committee of Southwest Hospital. Written informed consent was obtained from all participants.

### Image acquisition

The MRI experiment was performed using a 3-Tesla scanner (Magnetom Trio, Siemens, Erlangen, Germany) with a 12-channel phased-array head coil. DTI data were acquired using a single-shot twice-refocused spin-echo diffusion echo planar imaging sequence (repetition time = 10,000 ms, echo time = 92 ms, 64 non-linear diffusion directions with b = 1000 s/mm^2^ and an additional volume with b = 0 s/mm^2^, matrix = 128 × 124, field of view = 256 × 248 mm, 2 mm slice thickness without gap, 75 axial slices). T1-weighted three-dimensional magnetization-prepared rapid gradient echo (MP-RAGE) images were then collected using the following parameters: repetition time = 1,900 ms, echo time = 2.52 ms, inversion time = 900 ms, flip angle = 9°, matrix = 256 × 256, thickness = 1.0 mm, 176 slices with voxel size = 1 × 1 × 1 mm.

### Image processing

#### Morphological measurement of the CC

**Figure 1:**
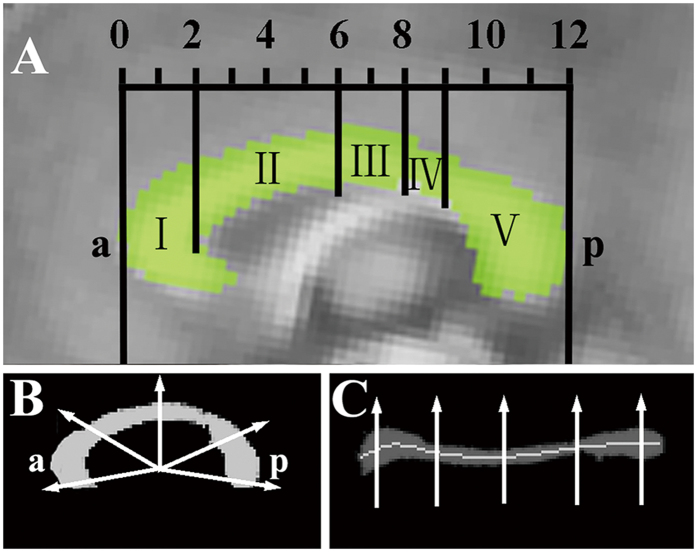
Segmentation and measurement of the CC in the mid-sagittal slice. (**A**) Geometric segmentation of the CC along its anterior (a) and posterior (p) extents. The scales indicate the length ratios of the five subregions, which connect the prefrontal areas, BA6, primary motor areas, primary sensory areas and posterior cerebral lobes, respectively. (**B**) Radial lines at 1.65° intervals (only five shown) were drawn from the midpoint of the anterior–posterior axis. (**C**) Radial lines intersecting the callosum were oriented vertically, and the median line (light gray) was defined as the median location of WM along each radial line.

Morphological measurement of the CC was performed using the C8 tool^25^, which automatically isolates the CC and measures callosal area and regional thickness based on WM segmentation derived from T1-weighted MRI data. The accuracy and reliability of C8 has been tested using two large imaging databases containing normal aging and dementia^25^. The parcellation of the CC was performed within standard Montreal Neurological Institute (MNI) space and then transformed back to native anatomical space by inverting the affine spatial transformation. Contiguous sets of WM voxels on the sagittal plane were selected and the boundaries of the callosal clusters were identified using a fixed WM segmentation threshold (0.55) in the C8 tool. The segmented CC was visually inspected for each participant. Thickness was computed at each point along the median CC line (Fig. 1) using the shortest line segment crossing the CC and passing through that point. The mean thickness within each subregion was then calculated, and area was defined as the total sum of the WM segmentation value. The mid-sagittal slice and one slice laterally on each side (x = ±1 mm in MNI space) were analyzed separately, and median values of the final derived quantities were used to increase the robustness of the overall procedure.

#### DTI data processing

The DTI data was pre-processed using FSL (University of Oxford, UK). First, the diffusion data were corrected for eddy currents and head motion. The images were masked to remove the skull and non-brain tissue using the FSL Brain Extraction Tool (BET)^26^. Then, diffusion parametric images were calculated using the diffusion tensor analysis toolkit (FDT)^27^. In addition, total brain volume (TBV), normalised for subject head size, was estimated with SIENAX^28^, which is part of the FSL.

#### Parcellation of the CC

The CC was segmented based on a classification method of five vertical CC partitions^10^. Specifically, region I connects the prefrontal areas; region II contains fibers that connect Brodmann area 6 (BA6), which is composed of the premotor cortex (PMC) and supplementary motor area (SMA)^29^; region III is composed of fibers that connect the primary motor areas; region IV connects primary sensory areas; and region V encompasses fibers that connect the posterior parietal, temporal, and occipital lobes (Fig. 1). For the DTI data, the non-diffusion-weighted (b0) volume was registered to the T1-weighted anatomical image with a six-parameter rigid body transformation and the inverted transformation was also acquired. Finally, median morphological measurements (callosal area and thickness) and DTI parameters (FA, AD, RD and MD) in each subregion were calculated for the three mid-sagittal slices in the original image space.

#### Probabilistic diffusion tractography (PDT)

**Figure 2:**
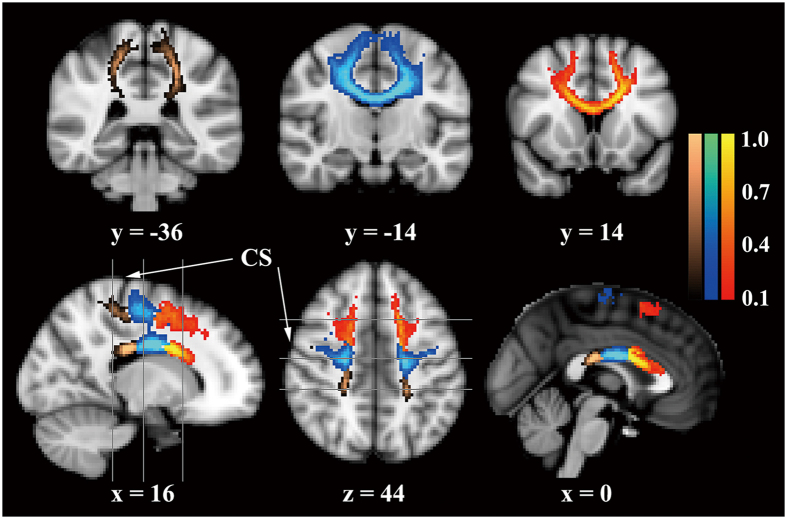
Maximum probability maps generated by tractography from the BA6, primary motor area and somatosensory area. The three transcallosal tracts are overlaid on the standard brain image with the MNI coordinate (x, y or z) and shown in red-yellow, blue-lightblue and copper, respectively. The color scales indicate the degree of overlap among all the subjects. Only voxels present in at least 10% of the subjects (eight subjects) are shown. The grey lines depict the locations of the three coronal planes (y = −36, −14 and 14, respectively). CS, central sulcus.

In order to verify our second hypothesis, six regions of interest (ROI) from the Juelich histological atlas^30^, including the PMC and SMA (BA6), the primary motor area (BA 4a and 4p) and the somatosensory area (BA 1, 2, 3a and 3b) in each brain hemisphere, were used as seed masks for multi-fiber probabilistic tractography^31^ in each subject’s native whole-brain image. For each participant, PDT was run from each voxel in the unilateral area and the contralaterally homogenous area was used as the target mask. The CC in the midsagittal slice was used as the waypoint mask to discard specious fibers. The tract masks were thresholded at a value equal to 40% of the 95th percentile of the distribution of the intensity values to correct for possible differences among tracts due to the different sizes of the ROIs^32^. The two tracts (left to right and right to left) were intersected to define the interhemispheric connecting fibers. The warpfields of nonlinear registration and the inverse versions were used for the translation between the original space and the standard space. The individual tracts were then binarized and summed across subjects to produce group probability maps for each pathway in the standard space. Finally, the three transcallosal tracts were classified by identifying the maximum of each voxel in the group probability maps to generate the maximum probability maps (Fig. 2), which can avoid the possible overlaps among the group probability maps. Individual mean values for DTI metrics (FA, AD, RD and MD) from the thresholded (>10%) maximum probability maps were then extracted from the normalized whole-brain DTI images, in which the voxels with FA value of <0.2 were discharged to avoid the contamination of non-WM.

### Statistical analysis

Differences in the demographic measurements were assessed using two sample t-tests, and the chi-squared test was used for gender. Then, multiple analyses of covariance were conducted to examine the differences in the multimodal measurements for each callosal subregion between the amputees and normal controls, using age, gender and TBV as covariates. A two-tailed *p* < 0.05 after Holm-Sidak correction for multiple comparisons was considered statistically significant. Finally, Spearman correlation analyses adjusted for age and gender were used to explore the associations between the clinical variables (amputation times and PLP levels) and imaging measurements across all patients. All statistical analyses were conducted using PASW software (version 17.0, Chicago, IL, USA).

## Results

### Demographic and clinical data

The demographic characteristics of the subjects are summarized in Table 1. There was no significant difference in age, gender, education level or MMSE score between the amputees and normal controls. The mean duration of time since amputation prior to the MRI scan was 42.6 ± 68.7 months. PLP was present in 14 patients and 9 patients were suffering from stump pain.

### Morphological differences in the CC

There were no statistically significant differences in mean callosal thickness or total area in the five subregions of the CC between the two groups (Table 2).

### Diffusion anisotropy differences in the CC

The amputees demonstrated significant larger values in AD, RD and MD in region II of the CC (*p* < 0.05, corrected, Table 3). There was also a trend towards lower FA in region II in the amputee group compared with the healthy controls (*p* = 0.07, corrected). Further 2 × 2 factorial analyses revealed no significant differences (*p* > 0.05, uncorrected) in DTI measures between amputees whose amputations occurred on different sides (left versus right) or at different sites (transfemoral versus transtibial).

### PDT

**Figure 3:**
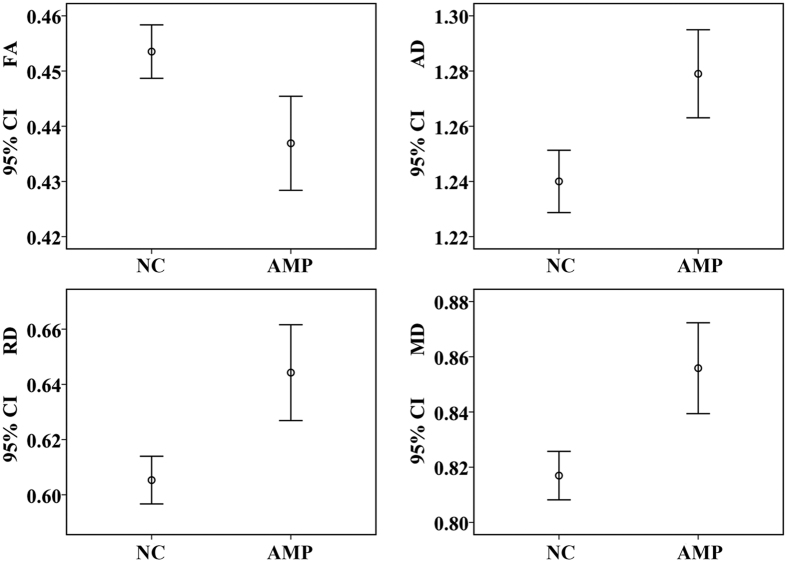
Differences in DTI measures for the fiber tracts connecting bilateral BA6 between amputees (AMP) and normal controls (NC). Axial diffusivity (AD), radial diffusivity (RD) and mean diffusivity (MD) values are ×10^−3^ mm^2^/s. FA, fractional anisotropy; CI, confidence interval.

Tractography from the seed masks confirmed that the trancallosal fibers cover the corresponding subregions of the CC (Fig. 2). Analyses of covariance (adjusted for age, gender and TBV) demonstrated that the mean FA value of the fibers connecting the bilateral BA6 in amputees was significantly smaller (*p* = 0.008), and the AD, RD and MD were larger (all the *p* values were <0.001, Fig. 3) relative to the normal controls. No other changes were significant for the fibers connecting the primary motor areas or the somatosensory areas after correction for multiple comparisons.

### Correlations between clinical variables and imaging measurements

Across all the amputees, partial correlation analyses revealed that the time since amputation was correlated with the FA value (ρ = −0.45, *p* = 0.006) and RD value (ρ = 0.37, *p* = 0.03) of region II. No other significant correlations were found between the imaging measurements of the CC and the clinical variables. To investigate the possible effects of the PLP, we further split the patients into two groups (amputees with PLP and amputees without PLP). Analyses of covariance (adjusted for age and the time since amputation) still failed to find any significant differences between the two groups.

## Discussion

Our findings identified microstructural variability within the CC after lower limb amputation. Smaller FA and significantly larger AD, RD and MD were found in region II of the CC in amputees compared with healthy controls. The tractography analysis also detected similar abnormalities in the fibers passing through region II of the CC, which link the bilateral PMC and SMA. In contrast, no macrostructural differences in total area or mean thickness were found in any subregion of the CC between the amputees and healthy controls.

The CC at the mid-sagittal plane can be discretely identified by conventional MRI and has been a target of extensive studies^14^,^33^,^34^,^35^,^36^. To distinguish segments of the CC, Hofer’s geometrical parcellation was used, which provides a clear description of the CC at the connectivity and functional levels^10^. In Hofer’s scheme, the topographic organization of the human CC is comprised of five segments with fibers projecting into different cortical areas. Compared with other common schemes, such as Witelson’s^37^ or Huang’s schemes^38^, Hofer’s strategy is more precise, as it accounts for a clear separation of callosal prefrontal, premotor and primary motor fiber bundles. Therefore, the current applied scheme is widely accepted in network and CC studies^39^,^40^,^41^. According to Hofer’s scheme, our findings point to abnormal WM integrity in region II of the CC, which supports our first hypothesis that CC subregion may be involved in brain reorganization following amputation.

We also used tractography in this study to identify alterations in the fiber tracts connecting the bilateral BA6. A possible explanation for these changes might be due to the function of these cortical regions. The SMA and PMC play an important role in inhibitory control and the planning of movement^42^. Using transcranial magnetic stimulation, previous research found that the PMC modulates the activity of contralateral motor areas during the preparatory period of a voluntary movement with the ipsilateral limb^43^,^44^. Such modulation is mediated by interhemispheric inhibition through fibers that cross the CC^45^,^46^, and enables healthy adults to perform complex motor tasks without the activation of contralateral muscles^47^. After unilateral lower limb amputation, the necessity of inhibiting the contralateral BA6 would be reduced. As a consequence, the fiber tracts could be altered due to functional disuse. In addition, the abnormalities in the WM connecting the bilateral PMC also offer a new explanation for the degradation of motor imagery in low limb amputees^48^, as previous studies revealed that functional connectivity between the bilateral PMC plays a critical role during motor imagery training^49^. Future studies are required to determine the precise impacts of these brain changes on clinical symptoms using behavioral tests.

Previous voxel-based studies across the whole brain have demonstrated FA changes in the body of the CC^4^ or the WM underlying the premotor cortex^21^ in lower limb amputees. As only nine amputees with phantom sensation were chosen in Simoes’s research, emphasis was given to the CC connecting the primary sensory areas^4^. In addition to the heterogeneity of the samples selected, the discrepancies in the reorganized subregions of the CC might also be attributed to limitation of the voxel-based statistical analysis, which is prone to be contaminated because of the spatial inconsistency of the reorganized brain regions if amputations occurred on different sides. By comparison with our previous study^21^, these results also support the widely accepted suggestions that although voxel-based analysis is rapid and fully automated, it is not a replacement for ROI-based analyses, as each has its own strengths and weaknesses, and could provide different types of information^50^. Voxel-based analysis can be used to generate hypotheses by identifying key areas that can subsequently be more thoroughly investigated using ROI approaches.

In both region II of the CC and the transcallosal fiber tracts of amputees, the FA values were smaller and other diffusion values were larger. In addition, the FA value and RD value in region II of the CC were correlated with the time since amputation. FA is highly sensitive to microstructural changes, but not very specific to tissue properties, such as axonal ordering, density and myelination^51^. High MD levels are likely related to increases in the spacing between membrane layers or increases in water content due to tissue inflammation or myelin loss in the brain^52^. RD has also been associated with myelination^53^. While animal studies link lower levels of AD to axonal damage^54^, human studies often associate higher AD with neurodegenerative processes, as in amyotrophic lateral sclerosis^55^, Alzheimer’s disease^56^ or chronic ischemia^57^,^58^. Therefore, the current pattern of integrity changes (reduced FA and increased AD/RD/MD) may reflect WM degeneration and myelin loss, which could be attributed to functional disuse. Nonetheless, the interpretation of changes to WM integrity is complicated due to complex fiber architecture, and thus should be done with caution. Similar abnormalities in DTI parameters were also detected in the CC of rats with traumatic brain injury, resulting in significant decreases in conduction of action potentials^59^. On the other hand, bilateral increases of interneuron activity after peripheral nerve damage were observed in rats^60^. Taken together, these reports imply that increased activation^4^,^5^,^6^ but reduced interhemispheric functional connectivity^7^ might be the cascade reaction following microstructural changes in the transcallosal pathway. Such speculation could be further investigated by follow-up observation on the therapeutic effectiveness induced by the modulation of the interhemispheric activity^18^.

The current study also raised a fundamental question concerning the discrepancies of reorganized brain regions between upper and lower limb amputations. Previous findings on the maladaptive reorganization of the sensorimotor cortex, especially in the primary motor area, were mainly based on upper limb amputation^5^,^6^,^61^,^62^,^63^,^64^. There are differences in the localization of representations for the upper and lower limbs in the sensorimotor cortex. Especially, increased complexity of hand movements resulted in a much broader representation in the primary motor area than the lower limb^65^. Besides, the extent or nature of cortical control of movement may also be more fundamentally different. For example, SMA activation was prominent for knee, but almost absent for elbow movement^66^, and brain activation associated with gait control was only found in the PMC and SMA^67^. By contrast, in agreement with our preliminary surface-based cortical thickness analysis^21^, the current findings supported that the reorganized brain regions following lower limb amputation were located at the premotor area (and the SMA) rather than the primary motor or somatosensory areas. The result was also supported by another MRI study on lower limb amputees using higher image resolution, which failed to find significant change of cortical thickness in the primary motor area^68^. Moreover, higher activation^4^ but lower volume^69^ in the BA6 was also reported in neuroimaging studies on lower limb amputation. The confounding effects of prosthesis use and PLP levels should also be mentioned here, as there may be associations between brain reorganization patterns and prosthesis^70^ or phantom pain^7^.

We measured mean callosal thickness and the total area of each CC subregion, and found no statistically significant differences between amputees and normal controls. Mid-sagittal CC demonstrates substantial inter-individual variability in local morphology including area and thickness among healthy individuals^71^,^72^, and previous studies showed that deprivation of sensory experience can modify the morphology of callosal fibers^73^, thus altering the communication between the two hemispheres. Therefore, it is necessary to evaluate the macrostructural difference in the CC between the two groups. The negative findings in the morphological analyses indicated that DTI is the modality of choice for the evaluation of the CC reorganization following amputation, and the diffusion abnormalities of the callosal fibers in amputees might not be attributed to the morphological variations.

Some limitations should be mentioned. First, it is impossible to differentiate input and output pathways in DTI data, so the current study cannot demonstrate a causative relationship between bilateral hemispheres. Multimodal brain imaging techniques could be used to invesigate the relationship between the dominant hemisphere and amputation side. Second, fiber bundles from the PMC and SMA cannot be partitioned due to the sensitivity of DTI to image resolution artifacts and crossing fibers. Consequently, the functional segregation between the PMC and SMA, such as planned and contingent motor control^74^, could not be further used to interpret the outcome of amputation in the current study. Third, the time course of WM reorganization following ampution could not be defined in the cross-sectional study. A previous study reported WM plasticity after 4 weeks of training^19^, and it is of interest to explore earlier WM alternations using longitudinal data. On the other hand, our findings could not completely rule out the possibility of macrostructural (volume or thickness) changes in WM of region II of the CC after a longer follow-up.

## Conclusion

We examined both morphological and diffusion changes in the CC of the same group of amputees for the first time. The thickness, area and DTI parameters were used to investigate five subregions of the CC. Diffusion alterations in region II of the CC were found in amputees compared with healthy controls. These changes suggest that fibers connecting the bilateral PMC and SMA are degenerating due to the degradation of motor coordination and imagery. The alterations are only reflected in DTI parameters, not morphological metrics, indicating that DTI is a more sensitive method of detecting brain reorgnization following amputation.

## Additional Information

**How to cite this article:** Li, Z. *et al*. Altered microstructure rather than morphology in the corpus callosum after lower limb amputation. *Sci. Rep.* **7**, 44780; doi: 10.1038/srep44780 (2017).

**Publisher's note:** Springer Nature remains neutral with regard to jurisdictional claims in published maps and institutional affiliations.

## Figures and Tables

**Table 1:** Demographic data of the participants.

Characteristics	Amputees (n = 38)	Controls (n = 38)	*p* value
Gender (male/female)	28/10	26/12	0.801
Age (years)	38.4 ± 13.2 (range: 18–60)	37.5 ± 12.2 (range: 19–60)	0.753
Education (years)	9.1 ± 4.0	9.4 ± 3.4	0.733
MMSE score	28.1 ± 1.4	28.2 ± 1.3	0.755
Time since amputation (months)	40.3 ± 68.0 (range: 1–336)	—	—
Amputation at left/right	16/22	—	—
Amputation at femur/tibia	16/22	—	—

The data are presented as the means ± standard deviation. MMSE, Mini-Mental Status Examination.

**Table 2:** Morphological measurements for the five callosal subregions.

Region	Metrics	Amputees	Controls	*p* value
I	Thickness	5.29 ± 0.98	5.66 ± 1.19	0.153
	Area	144.50 ± 25.54	142.32 ± 26.65	0.811
II	Thickness	5.19 ± 0.80	5.35 ± 0.97	0.522
	Area	149.29 ± 26.50	160.42 ± 45.18	0.202
III	Thickness	4.74 ± 095	5.03 ± 1.26	0.258
	Area	53.79 ± 12.34	71.94 ± 92.55	0.233
IV	Thickness	4.15 ± 1.05	4.69 ± 2.42	0.215
	Area	25.05 ± 7.70	28.94 ± 14.21	0.162
V	Thickness	5.52 ± 1.49	5.74 ± 2.08	0.660
	Area	175.58 ± 30.29	173.97 ± 29.42	0.796

The units for thickness and area are mm and mm^2^, separately. Thickness was calculated as the mean thickness within a geometrically-defined subregion. Note that the *p* value was not corrected for multiple comparisons.

**Table 3:** DTI measurements for the five callosal subregions.

Region	DTI metrics	Patients	Controls	*p* value
I	FA	0.61 ± 0.04	0.62 ± 0.02	0.621
	AD	1.67 ± 0.11	1.66 ± 0.06	0.835
	RD	0.59 ± 0.08	0.57 ± 0.03	0.809
	MD	0.95 ± 0.08	0.93 ± 0.04	0.857
II	FA	0.48 ± 0.03	0.50 ± 0.02	0.07
	AD	1.74 ± 0.14	1.66 ± 0.06	0.037*
	RD	0.87 ± 0.11	0.81 ± 0.05	0.035*
	MD	1.16 ± 0.12	1.09 ± 0.05	0.020*
III	FA	0.48 ± 0.04	0.48 ± 0.03	0.932
	AD	1.82 ± 0.18	1.73 ± 0.09	0.113
	RD	0.88 ± 0.15	0.82 ± 0.07	0.354
	MD	1.19 ± 0.15	1.13 ± 0.07	0.202
IV	FA	0.45 ± 0.05	0.47 ± 0.05	0.731
	AD	1.90 ± 0.16	1.84 ± 0.12	0.584
	RD	0.95 ± 0.15	0.89 ± 0.12	0.568
	MD	1.27 ± 0.15	1.21 ± 0.11	0.576
V	FA	0.66 ± 0.02	0.68 ± 0.02	0.238
	AD	1.88 ± 0.09	1.87 ± 0.11	0.925
	RD	0.61 ± 0.06	0.58 ± 0.04	0.246
	MD	1.04 ± 0.06	1.01 ± 0.05	0.603

Single star (*) represents the difference is significant at *p* < 0.05 (Holm-Sidak correction for multiple comparisons). Axial diffusivity (AD), radial diffusivity (RD) and mean diffusivity (MD) values are ×10^−3^ mm^2^/s. FA, fractional anisotropy.
